# Behavior and Functional Roles of CD34^+^ Mesenchymal Cells in Mammalian Testes

**DOI:** 10.3390/ijms23179585

**Published:** 2022-08-24

**Authors:** Shin-ichi Abe

**Affiliations:** Faculty of Health Science, Kumamoto Health Science University, Kumamoto 861-5598, Japan; abe@kumamoto-hsu.ac.jp

**Keywords:** testes, mammals, interstitium, mesenchymal cells, telocytes, Leydig cell, CD34, PDGFRα, 3D re-aggregate culture

## Abstract

Mammalian testes consist of seminiferous tubules within which Sertoli cells line up at the periphery and nurse germ cells, and of interstitia that harbor various cells such as peritubular myoid cells (PMCs), Leydig cells (LCs), vascular endothelial cells, immune cells such as macrophages, and mesenchymal (stromal) cells. Morphological studies have recently reported the presence of telocytes with telopodes in the interstitium of adult mouse, rat, and human testes. CD34^+^PDGFRα^+^ telocytes with long and moniliform telopodes form reticular networks with various cell types such as LCs, PMCs, and vessels, indicating their potential functions in cell–cell communications and tissue homeostasis. Functional studies have recently been performed on testicular interstitial cells and CD34^+^ cells, using 3D re-aggregate cultures of dissociated testicular cells, and cell cultures. Direct observation of CD34^+^ cells and adult LCs (ALCs) revealed that CD34^+^ cells extend thin cytoplasmic processes (telopodes), move toward the LC–CD34^+^ cell-re-aggregates, and finally enter into the re-aggregates, indicating the chemotactic behavior of CD34^+^ telocytes toward ALCs. In mammalian testes, important roles of mesenchymal interstitial cells as stem/progenitors in the differentiation and regeneration of LCs have been reported. Here, reports on testicular telocytes so far obtained are reviewed, and future perspectives on the studies of testicular telocytes are noted.

## 1. Development of Testes

Testes develop from the bipotential gonad that otherwise develops into ovaries, in mammals [1,2,3]. *Sry* gene expression in the embryonic gonad harboring the Y chromosome directs the differentiation of Sertoli cells which orchestrate germ cells and other cells to proceed through many successive steps toward sperm formation. Pre-Sertoli cells aggregate and surround germ cells, defining testis cords and interstitial tissues, due to partitioning by vascular endothelial cells that immigrate from the mesonephros. Peritubular myoid cells (PMCs) differentiate around the cords, which then elongate to form seminiferous tubules. Fetal Leydig cells (FLCs) arise in the interstitial area of the fetal testes and increase in number, but they begin to decrease after birth, yet they persist even in adults [4,5,6]. On the other hand, adult Leydig cells (ALCs) appear during the pubertal period, and increase in number. ALCs are different from FLCs in morphology, developmental processes, and functions [4,5,6,7,8,9,10]. Both of them have important roles in androgen secretion that propel spermatogenesis and masculinization. However, in contrast to ALCs, FLCs lack 17β-hydroxysteroid dehydrogenase type 3 (HSD17β3), an enzyme required for the final reaction of testosterone synthesis, so FLCs cannot produce testosterone, while Sertoli cells convert androstenedione produced by FLCs into testosterone in the fetal stage [11]. ALC development proceeds in multiple steps from the precursor cells to progenitors, immature ALCs, and mature ALCs [4,5]. During the steps from the spermatogonial stem cells toward sperm formation, many cellular interactions, hormones, and growth factors are involved, and many excellent reviews thereof have been published [1,2,3,12]. In the current review, I will focus on the theme of interstitial cells, especially mesenchymal CD34^+^ cells and telocytes.

Testis interstitium harbors steroidogenic LCs, PMCs, macrophages, vasculature, pericytes, and mesenchymal cells (fibroblasts) [13]. Testicular interstitial cells in the fetal gonad derive from the coelomic epithelium and mesonephros [14,15]. The coelomic epithelium generates the supporting and interstitial progenitor cell lineages; the former differentiates into Sertoli cells, and the latter into FLCs in response to hedgehog signaling that is conferred by Sertoli cells, and into non-steroidogenic interstitial cells [15]. A population of the non-steroidogenic interstitial cells differentiate into ALCs in the postnatal testes. The mesonephros produces a population of cells that migrate into the gonad, and most of them differentiate into vascular endothelial cells, while the rest differentiate into FLCs independently of hedgehog signaling [15]. On the other hand, ALCs are thought to arise from stem/progenitor cells that reside at peritubular positions of seminiferous tubules and at perivascular niches [16]. Another possibility that has been presented is that ALCs originate from FLCs that undergo dedifferentiation, followed by transdifferentiation into ALCs, in the mouse [17] and primate [8]. The extent to which each possibility contributes to ALC differentiation remains to be clarified. Platelet-derived growth factor (PDGF) signaling through the receptor PDGFRα plays an important role in testis formation and differentiation of FLCs and ALCs from their progenitors [18,19]. In the mouse XY gonad, PDGFRα is expressed in the region near the coelomic epithelium at 12.5-dpc, and in the interstitial cells and PMCs surrounding the seminiferous cords at 13.5-dpc and 17.5-dpc, as well as in the interstitium of pubertal and adult testes [20,21]. Abe et al. [22] also examined the expression of PDGFRα in embryonic and postnatal mouse testes. PDGFRα is expressed in the interstitium, but not in CD31^+^ endothelial cells or in the coelomic vessel at 13.5-dpc. At 17.5-dpc, and in neonatal, pubertal, and adult testes, PDGFRα is expressed throughout the interstitial cells, but not in the endothelial cells.

Kuroda et al. [23] reported the presence of CD34^+^ stromal cells in the fetal and adult human testes, where the CD34^+^ cells and α-smooth muscle actin^+^ myofibroblasts are located in the outer and inner layers of the peritubular tissue, respectively. The CD34^+^ stromal cells extend thin cytoplasmic processes to form a reticular network with macrophages and LCs. Although the authors did not designate these cells as ‘telocytes’ (because it was not until 2010 that Popescu and Faussone-Pellegrini [24] called a novel type of interstitial cell (Cajal-like) ‘telocytes’), their findings indicated for the first time the presence of ‘telocytes’ in vertebrate testes. Thereafter, there have been some reports that described intertubular stromal cells as ‘telocytes’ in Chinese soft-shelled turtle, *Pelodiscus sinensis* [25], human [26], rat [27], and mouse [28]. All of the above four papers reported that cells located in the interstitial stroma display CD34 and PDGFRα, and show the typical shape of telocytes, with long and moniliform projections (telopodes). The telocytes form reticular networks with various cell types such as themselves, LCs, and PMCs; and with vessels, indicating potential functions in cell–cell communications and tissue homeostasis. In many organs besides testes, CD34, PDGFRα, c-kit, and vimentin have been reported as markers of telocytes [29]. Pawlicki et al. [28] reported that telocytes express c-kit and vimentin in mouse testes. However, c-kit is expressed in LCs and differentiating type A spermatogonia in mouse [30,31], and vimentin, an intermediate filament, is mainly expressed in Sertoli cells [32]. Thus, neither c-kit nor vimentin seem to be appropriate for a marker of telocytes in testes. All of the above four papers [25,26,27,28] reported on testes only from adults, so Abe et al. [22] assessed the distribution of CD34 and PDGFRα in testes from fetal through adult mice. At 13.5-dpc, CD34 is expressed only in CD31^+^VE-cadherin (VE-cad)^+^ endothelial cells that have long extensions and that are distributed in the mesonephros, the interstitium of the testis and the coelomic vessel. At 17.5-dpc, CD34^+^ cells are identical with CD31^+^VE-cad^+^ single cells scattered in the interstitum, and CD31^+^VE-cad^+^ cells comprising vasculature. After birth, CD34 expression starts newly in the interstitial areas where CD31 is not detected, while CD34 is continually expressed with CD31 and VE-cad in the endothelial cells. These expression patterns of CD34, CD31, and VE-cad continue through adulthood. PDGFRα is displayed broadly in the interstitial cells, but not in CD31^+^ endothelial cells, throughout development from the fetal to adult testes. Thus, it seems to be reasonable to assume that CD34^+^PDGFRα^+^ cells include telocytes in the mouse testes throughout the development.

## 2. Roles of Interstitial Cells in the Reconstruction of Testicular Structures in 3D Re-Aggregate Culture

In order to analyze the mechanisms underlying the morphogenesis and maintenance of the testicular structures, differentiation of germ and somatic cells, and causes of male infertility, trials to establish testis organoid models that recapitulate complete spermatogenesis have been reported by many investigators [33,34,35,36]. Many approaches using various scaffolding biomaterials such as agarose, collagen, etc., species, ages, cell sources, and scaffolding strategies have been applied to make organoid models, but few studies have shown any clear evidence of success in the reconstruction of seminiferous tubule-like structures within which germ cell differentiation proceeds. Zhang et al. [37] succeeded in the reconstruction of testicular structures via 3D re-aggregate culture of dissociated testicular cells from neonatal mouse testes within a collagen matrix. KnockOut Serum Replacement (KSR) is essential for the reconstruction of seminiferous tubule-like structures with a lumen, within which polar Sertoli cells align and bind with each other via tight junctions and hold germ cells, and outside of which PMCs surround the tubules and LCs reside with interstitial cells in the interstitium. Sertoli cells differentiate and express androgen receptors. Spermatogonia proliferate and differentiate into primary spermatocytes, and even after three weeks, PLZF-positive undifferentiated spermatogonia maintain proliferative activity [37]. In the absence of KSR, no ordered structures defining tubule-like structures and interstitial tissues are formed. Sertoli cells re-aggregate on their own, but form no ordered re-aggregates. The proliferative activity of spermatogonia remains at a low level, and spermatogonia do not differentiate into primary spermatocytes. Although complete spermatogenesis into mature sperm has not yet been accomplished using this in vitro system, it seems to be one of the excellent systems for the analysis of the mechanism underlying cell-to-cell interactions and morphogenesis in spermatogenesis [37]. The significance of KSR in the reconstruction was confirmed by Zhang et al. [38].

In order to investigate the behaviors and roles of CD34^+^ cells and PMCs in the reconstruction of seminiferous tubule-like structures, Abe et al. [39] observed the behavior of CD34^+^ cells, p75^+^ cells, and PMCs in re-aggregate cultures. CD34^+^ cells, p75^+^ cells, and Sertoli cells initially exist as re-aggregates on their own, while most PMCs are distributed as single cells. While being cultured for one week, CD34^+^ cells, p75^+^ cells, and PMCs mix with each other, and then they segregate into three layers in a re-aggregate; PMCs, p75^+^ cells, and CD34^+^ cells become localized in the outermost, middle, and inner layer, respectively, in a re-aggregate of the three cell types. On the other hand, re-aggregates of Sertoli cells enlarge and the nuclei are situated at the periphery of the re-aggregates to form seminiferous tubule-like structures with lumens. The Sertoli cell layer is surrounded by PMCs as in vivo. The three-layer-structure of PMCs, p75^+^ cells, and CD34^+^ cells in the interstitium is also very similar to the peritubular and interstitial tissues in vivo. Live imaging of the reconstruction process in re-aggregate cultures using *Sox9-EGFP* mice, in which Sertoli cells are labeled by EGFP, shows that Sertoli cells and non-fluorescent testicular cells gradually segregate to form fluorescent re-aggregates of Sertoli cells and dark re-aggregates of non-fluorescent cells. These results indicate that Sertoli cell-re-aggregates assemble to form a cord-like structure, while CD34^+^ cells, p75^+^ cells, PMCs, and other cells such as LCs re-aggregate and segregate to form interstitium-like structures [39]. To examine the role of the interstitial and peritubular cells in the reconstruction of seminiferous tubule-like structures, re-aggregate cultures deprived of those cells have been performed, resulting in a failure of the reconstruction of seminiferous tubule-like structures. When the interstitial and peritubular cells are added back to the re-aggregate cultures lacking those cells, seminiferous tubule-like structures are reconstructed [39]. These results indicate that interstitial and peritubular cells are indispensable for the reconstruction of the seminiferous tubule-like structures from dissociated cells derived from neonatal mouse testes. What type(s) of cells in the interstitium, CD34^+^PDGFRα^+^ cells, LCs, PMCs, immune cells, and/or endothelial cells, is/are indispensable for the reconstruction of the seminiferous tubule-like structures remain to be clarified. Our group has also shown that when Alk5i (activin receptor-like kinase 5 inhibitor) is applied to cultures of testicular re-aggregates, the reconstruction of the seminiferous tubule-like structures is disturbed, indicating that TGFβ signaling through ALK5 is indispensable for the reconstruction of cord-like and tubule-like structures [39]. Miles et al. [40] showed that when ALK5i is added to 11.5-dpc testis with mesonephros and 12.5-dpc testis-only samples in culture, normal testis cord formation is disrupted. ALK5 is expressed in somatic cells and germ cells in embryonic testes [40]. Abe et al. showed that CD34^+^ cells purified from 10-dpp mouse testes differentiate into α-smooth muscle actin^+^ PMCs through p75^+^ cells in cell culture [39]. The addition of Alk5i during the culturing of CD34^+^ cells disturbs their differentiation into PMCs, and also affects their shape. These results indicate that Alk5i works directly on CD34^+^ cells, and that CD34^+^ cells play important roles in the reconstruction of the seminiferous tubule-like structures. It seems that it will be necessary to find out in which cell type(s) ALK5 is expressed, and how ALK5i disturbs the reconstruction of the seminiferous tubule-like structures.

## 3. Interaction between CD34^+^ Telocytes and Other Testicular Cells In Vitro

**Figure 1:**
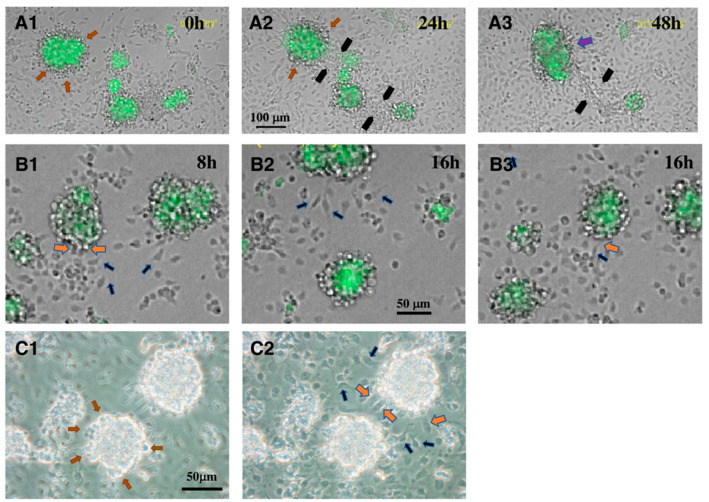
Live cell imaging shows direct interaction between CD34^+^ cells (telocytes) and ALCs in culture. (modified from Figure 4 and Supplementary Figure S3 in Abe et al. [41]). (**A1**–**A3**,**B1**–**B3**) Merged figures of GFP (ALCs) and bright-field (CD34^+^ cells) images are shown. All photographs shown here are slice snapshots. (**A1**–**A3**) An example showing that two re-aggregates, each consisting of LCs (inside) and CD34^+^ cells (outside) are connected by cytoplasmic processes extending from CD34^+^ cells on each re-aggregate (black arrows in (**A2**,**A3**)) and coalesced into one re-aggregate (purple arrow in (**A3**)). Orange arrows in (**A1**,**A2**) show round CD34^+^ cells that attach around LC-re-aggregates. (modified from Figure 4C1–C3 [41]). (**B1**–**B3**) Behavior of CD34^+^ cells toward re-aggregates of LCs and CD34^+^ cells. Blue arrows in (**B1**,**B2**) show CD34^+^ cells that attach to the dish, extend filopodia (telopodes), and are approaching re-aggregates of LCs and CD34^+^ cells. Orange arrows in (**B1**,**B3**) show CD34^+^ cells that are just plunging into re-aggregates of LCs and CD34^+^ cells. (modified from Figure 4B2,B3 [41]) (**C1**,**C2**) Phase contrast microscopy shows re-aggregates of LCs and CD34^+^ cells, and CD34^+^ cells that attach to the dish. (**C1**) Focus is on some round CD34^+^ cells (orange arrows) that attach around LC re-aggregates. (**C2**) Focus is on CD34^+^ cells that attach to the dish. Blue arrows indicate CD34^+^ cells that are approaching re-aggregates of LCs and CD34^+^ cells by extending thin filopodia (telopodes). Orange arrows show CD34^+^ cells that are just plunging into re-aggregates of LCs and CD34^+^ cells. (modified from Supplementary Figure S3 [41]).

There have been few reports demonstrating direct interactions between CD34^+^ telocytes and other surrounding cell types, or analyzing the mechanism underlying the interactions. Abe et al. [41] reported on the interaction between CD34^+^ telocytes and LCs in vitro. In a re-aggregate culture of dissociated testicular cells, ALCs are incorporated, such as in vivo, within the re-aggregates of CD34^+^ cells that occupy the major part of the interstitial-like structures, whereas FLCs are not. There are at least two possible scenarios for this phenomenon. One is that ALCs actively move into the re-aggregates of CD34^+^ cells. The other is that CD34^+^ cells move toward ALCs and incorporate them into the re-aggregates of CD34^+^ cells. Both ALCs and CD34^+^ cells express integrins α4, α9, and β1, and VCAM1 (one of the ligands for integrins α4β1 and α9β1), but FLCs barely do. Integrin β1 is also expressed in PMCs and in some Sertoli cells, while integrins α4 and α9 are weakly displayed in PMCs, and barely in Sertoli cells and germ cells. VCAM1 is barely expressed in Sertoli cells, PMCs, and germ cells. The addition of function-blocking antibodies against integrin α4, α9, and β1, and against VCAM1, disturbs the reconstruction of the interstitial tissue-like structures and the seminiferous tubule-like structures, indicating that integrins α4β1, α9β1, and VCAM1 are involved in the normal reconstruction of the interstitial tissue-like structures, as well as seminiferous tubule-like structures, and that ALCs and/or CD34^+^ cells play a critical role in the reconstruction. The authors also showed that antibodies against integrins α4 and β1, and VCAM1 block the re-aggregation of purified CD34^+^ cells and their binding to VCAM1, indicating that CD34^+^ cells adhere to themselves through α4β1 integrin and VCAM1 [41]. One of the most intriguing findings in that report were the apparently direct interactions between CD34^+^ cells and ALCs in culture. When CD34^+^ cells and GFP^+^ALCs, each of which has been purified using FACS, are put together in a culture dish, the two cell types make round re-aggregates in which LCs are localized inside of the aggregates, and CD34^+^ cells outside (Figure 1A1,C1). When two re-aggregates come across each other, the CD34^+^ cells extend many thin cytoplasmic processes (probably telopodes) and connect to the processes extending from the other re-aggregate (black arrows in Figure 1A2,A3), and finally merge into one aggregate (purple arrow in Figure 1A3). On the other hand, CD34^+^ cells that attach to the bottom of the dish extend their cytoplasm soon after inoculation (Figure 1B1). Some of them extend thin cytoplasmic processes (filopodia, telopodes), and move toward the LC–CD34^+^ cell aggregates, while swaying their filopodia (blue arrows in Figure 1B1,B2,C2). Touching the aggregates with the filopodia, the CD34^+^ cells finally enter into the aggregates (orange arrows in Figure 1B1,B3,C2) (also refer to Supplementary Video S2 in [41]). These behaviors of the CD34^+^ cells are not observed toward aggregates consisting of only CD34^+^ cells, indicating that CD34^+^ cells move specifically toward LCs by chemotaxis (or haptokinesis). This phenomenon is suppressed by the treatment of LCs and CD34^+^ cells with the VCAM1 antibody, indicating that VCAM1 plays an important role in the chemotaxis (or haptokinesis) of CD34^+^ cells toward LC–CD34^+^ cell aggregates. VCAM1 is a cell adhesion molecule involved in the regulation of inflammation [42,43]. Soluble VCAM1 (sVCAM1) shows chemotactic activity toward T cells (Jurkat T cells and IL-2-dependent T cells) which have high affinity VLA-4 (α4β1 integrin) [44]. Tumor necrosis factor alpha (TNFα), a pro-inflammatory cytokine, induces the expression of VCAM1 in endothelial cells and other cells. α4β1 integrin, expressed on leukocytes such as macrophages and T cells, directly binds with VCAM1 on the endothelial cells, which in turn activates VCAM1, and through signal transduction pathways, results in the weakening of the cell–cell adhesion, leading to the promotion of leukocyte transendothelial migration [43]. These results strongly indicate that, although moniliform has not been confirmed on the cytoplasmic processes due to the limit of resolution of the light microscope used, the CD34^+^ cells that we have observed are telocytes with long telopodes extending toward LCs with active motion. The results also indicate that the CD34^+^ telocytes have locomotive activity and show a protean nature, taking on different forms depending on their environment. They do not always show telopodes, which may be extended when they detect some signals from other cell types and move toward them (in this case, signals from ALCs). In the differentiation of ALCs, as well as in their regeneration in ethane dimethane sulfonate (EDS)-treated rats in vivo, it is thought that mesenchymal stem/progenitor cells (very probably CD34^+^ cells) start to proliferate at a peritubular position, and that the progenitor cells and/or immature cells move toward the central interstitium, making clusters, and finally differentiate to mature ALCs [5]. Eventually, mature ALCs are enclosed by the thin cytoplasmic extensions of the CD34^+^ telocytes. Thus, the observations reported by Abe et al. [41] suggest that CD34^+^ telocytes play a critical role in the differentiation and regeneration of ALCs in vivo. In addition, Abe et al. reported that the integrins α4, α9, and β1 are expressed in the interstitium of embryonic mouse testes [22], and DeFalco et al. [14] reported that VCAM1 is exhibited in cells accompanying endothelial cells that migrate from mesonephros into male gonads. These findings raise the interesting question of what roles VCAM1–integrin α4β1 and/or VCAM1–integrin α9β1 interactions play in the embryonic testes.

## 4. Roles of CD34^+^ Mesenchymal Cells as a Constituent of Stem Cell Niche and/or Stem/Progenitor Cells

In general, CD34^+^ mesenchymal/stromal cells are considered to play crucial roles in tissue renewal and regeneration as a constituent of a stem cell niche in various organs such as intestine, skeletal muscle, heart, lung, and skin [24,45,46,47,48,49,50,51,52]. Two functions of CD34^+^ mesenchymal cells in various stem cell niches are reported; one is in support of the proliferation of stem cells and their differentiation during regeneration, and the other is as progenitors of differentiated cells in the regeneration in response to inflammation. For example, during the repair of the enteric wall, tissue-resident human CD34^+^ mesenchymal cells play a role as a source of α-smooth muscle actin^+^ myofibroblasts [52]. As mentioned in an earlier section, two types of LCs, FLCs and ALCs, develop in the fetal and postnatal testes, respectively. A model of two pathways of FLC differentiation has been proposed [15,53]. The first pathway is that progenitors are derived from the coelomic epithelium, and differentiate into FLCs directly; the second one is that progenitors are derived from Nestin-positive perivascular cells that co-migrate with endothelial cells from the mesonephros into the testis. Nestin is an intermediate filament protein and a marker for stem/progenitor cells in various tissues such as neural tissue [54] and bone marrow [55,56]. Nestin^+^ perivascular progenitors in the fetal testis have been shown to be multipotent cells that can generate LCs, pericytes, and PMCs. They are maintained in an undifferentiated state by an active Notch signal, which is conferred by blood vessels, and differentiates upon its loss [53]. Nestin^+^ cells co-express ARX (Aristaless related homeobox gene) and NR2F2 (COUP-TFII (Chicken Ovalbumin Upstream Promoter-Transcription Factor II)). *Arx* is expressed in various tissues and organs, including the brain and gonad of mouse fetuses, and its mutants show abnormal development of brain and testis in mouse and human [57]. In *ARX* KO testes, the number of FLCs markedly decreases, due to the low proliferative activity of their progenitors [58]. Based on the expression pattern, ARX is thought to be a marker of progenitors for FLCs. COUP-TFII is a member of the nuclear receptor superfamily that is highly expressed in mesenchyme and plays indispensable roles during mouse development [59]. None of the Nestin, ARX, or NR2F2 are expressed in PECAM1 (CD31)^+^ endothelial cells [53]. On the other hand, CD34 is expressed only in CD31^+^VE-cad^+^ endothelial cells, some of which are scattered in the interstitum, and the rest of which comprise the vasculature in mouse fetal testes [22]. This indicates that CD34 is not displayed in the Nestin^+^ARX^+^NR2F2^+^ cells in the embryonic testes. Whether telocytes are present in the fetal testes or not remains unknown.

On the other hand, extensive efforts have been devoted to identify stem cells/progenitors of ALCs, and many candidates for the markers have been reported in rodents and human [9]. To find stem cells/progenitors for ALCs, EDS is injected intraperitoneally into adult rat and mouse, which eliminates ALCs of the testes and induces the proliferation of some mesenchymal cells on the seminiferous tubules and their differentiation into LCs that produce testosterone [60]. Davidoff et al. [61] reported that LCs regenerate within two weeks after EDS injection as cell clusters in the vicinity of vessels, and as single, spindle-shaped cells located at the peritubular position. After about a month, the cells are mainly accumulated within the intertubular connective tissue. Candidates for the stem cells of ALCs (SLCs) were suggested to be Nestin^+^ vascular pericytes [61], and the authors concluded from the results of immunostaining experiments that Nestin^+^ stem cells stick out from the vessel walls and regenerate LCs as cell clusters in their vicinity. Ge et al. [62] purified putative SLCs from neonatal rat testes via the selection of 3β-HSD^−^LHR^−^PDGFRα^+^ interstitial cells, based on the fact that PDGFRα expression is restricted to the LC lineage. Cultures of these cells showed continued proliferation and differentiation with the expression of 3β-HSD and the production of androgen in differentiation-inducing medium. When fluorescently labeled LHR^−^PDGFRα^+^ interstitial cells were transplanted into EDS-treated testes, some of them differentiated into 3β-HSD^+^ cells. Furthermore, in cultures of the seminiferous tubules separated from EDS-treated testes, 3β-HSD^−^ cells proliferate on the tubule surfaces and some of them differentiate into 3β-HSD^+^ LCs, which produce testosterone [62]. Repeated treatment of the cultured tubules with EDS, and culturing of the tubules resulted in the reappearance of testosterone-producing cells, suggesting that the PDGFRα^+^ peritubular non-steroidogenic mesenchymal cells are stem cells for newly formed LCs [63]. Qin et al. [64] showed that COUP-TFII is highly expressed in mesenchymal cells in embryonic and early postnatal testes, and the conditional deletion of the *Coup-tfII* gene by Cre-ER recombinase in prepubertal male mice resulted in the failure of ALC development, indicating the important role of COUP-TFII for progenitor LC maturation. Kilcoyne et al. [65] showed that ALCs are derived from COUP-TFII^+^ cells, which are putative SLCs, based on findings in ablation/regeneration models produced using the EDS treatment of rats and mice. COUP-TFII^+^ SLCs are present in the fetal testes of humans, marmosets, and rodents. PDGFRα is occasionally expressed in COUP-TFII^+^ stem cells during the first week after EDS treatment. Jiang et al. [66] showed that cells that express GFP under the *Nestin* promoter in the neonatal mouse testicular interstitium have an extensive ability to proliferate in culture. The *Nes*-GFP^+^ cells differentiate into several lineages, such as neural, osteogenic, adipogenic, and chondrogenic cells, as well as LCs with 3β-HSD expression and testosterone production in a differentiation-inducing medium. The transplantation of the *Nes*-GFP^+^ cells into adult mouse testes treated with EDS results in their differentiation into LCs that colonize the interstitium [66]. A fairly high percentage (70–90%) of the *Nes*-GFP^+^ cells derived from 7-dpp testes express leukemia inhibitory factor receptor (LIFR), PDGFRα, CD51, and p75/neurotrophin receptor (NTR) [66]. Zang et al. [67] reported that CD51 (integrin αv) is expressed in the interstitial cells of postnatal mouse testes, and the CD51^+^ cells isolated from adult testes undergo active proliferation and differentiation into multiple mesenchymal cell lineages and LCs in culture when appropriate chemicals are added. CD51^+^ cells transplanted into the testes of EDS-treated rats differentiate into mature LCs and produce testosterone. CD51^+^ cells express some SLC markers so far reported, such as Nestin, PDGFRα, and LIFR [67]. Our group reported that most of the CD34^+^CD31^−^ interstitial cells in postnatal mouse testes express PDGFRα (until they become adults) and p75 (until puberty) [22]. Thus, it seems reasonable to assume that a high percentage of CD34^+^CD31^−^ interstitial cells display Nestin, CD51 and LIFR, though a quantitative estimation of the extent of overlap is unknown. Based on microarray data from rat testes, CD90 (Thy-1) was found to be another candidate for an SLC marker [68]. CD90^+^ cells isolated from adult testes proliferate and differentiate into LCs in culture with desert hedgehog (DHH), and produce testosterone. It is unknown whether CD90 is expressed in CD34^+^CD31^−^ interstitial cells. On the other hand, in adult human testes, p75^+^ cells express an SLC marker, Nestin (immunostaining shows almost complete overlap), but not HSD3β [69]. The p75^+^ cells purified by flow cytometry from adult human testes can be expanded in culture and show the potential for multi-lineage differentiation into osteogenic, adipogenic, and chondrogenic lineages, as well as testosterone-producing LCs. When the cells are transplanted into EDS-treated rat testes, the p75^+^ cells differentiate into LCs in vivo and secrete testosterone.

Recently, some attempts to decipher cell lineage specification during spermatogenesis using single-cell RNAseq (sc-RNAseq) have been reported. Stevant et al. [70] identified two clusters of interstitial progenitors, both of which express *Coup-TFII*, *Pdgfrα*, and *Tcf21* (a basic helix-loop-helix transcription factor) in the embryonic testis from *Nr5a1(Ad4BP)-GFP* mouse. Green et al. [71] also found a cluster of mesenchymal cell population expressing *Tcf21*, *Col1A1*, etc., which is distinct from both PMCs and LCs, in adult mouse testicular cells. Shen et al. [72] showed that the *Tcf21*^+^ cell population isolated from adult mouse testes is heterogeneous, and tSNE analysis identified five Tcf21^lin^ clusters, of which three express CD34. Immunostaining showed that the *Tcf21*^+^ cell population is localized in the interstitium surrounding the seminiferous tubules, and that the expressions of Tcf21 and CD34 overlap to some extent, but do not accord completely with each other in adult testes [72]. Their results indicate that the CD34^+^ cell population is heterogeneous. Interestingly, the fractionated *Tcf21*^+^ cells are able to differentiate into either PMCs or LC lineages in vivo and in vitro, indicating that they are multipotent progenitors with mesenchymal stem cell-like properties. CD34^+^ cells purified from 10-dpp mouse testes also differentiate into α-smooth muscle actin^+^ PMCs through p75^+^ cells in cell culture [39]. Since the co-expressions of Tcf21 and CD34 throughout developmental stages have not been studied, the relationship between Tcf21^+^ cells and CD34^+^ cells remains to be clarified. To find and characterize the SLC population, Guan et al. [73] analyzed by sc-RNAseq the mesenchymal cell populations that show proliferative activity (probably including SLCs) after the EDS treatment of adult rats. They identified by transcriptomic analysis five stromal clusters that can be classified into two major groups: proliferation and differentiation populations, the former of which may represent stem cells. The authors concluded that Nestin and CD90 may be better markers for SLCs, compared to the other ones previously reported (PDGFRα, NR2F2, ARX, Tcf21, NGFR/p75, CD51, and Gli1), because cells expressing Nestin and CD90 match those expressing cell division genes.

Thus, more than a few lines of evidence support the presence of stem cells/progenitors, not only for LCs, but also for mesenchymal cells such as osteogenic, adipogenic, and chondrogenic cells in the interstitium of rodent and human testes. Many candidates for markers for the stem cells have been proposed, and some of the cells expressing them seem to include CD34^+^ cells. However, the markers essential for the isolation of the stem cells/progenitors do not seem to have been clearly specified. Moreover, there may be a species-specific problem concerning stem cell markers. For example, CD51^+^ cells isolated from adult mouse testes have the potential to differentiate into multiple mesenchymal cell lineages and LCs in culture, and when transplanted into EDS-treated rat testes, they differentiate into mature LCs [67]. In adult human testes, however, HSD3β^+^ mature LCs express CD51 [69]. Thus, CD51 is not appropriate for a candidate of a human SLC marker. On the other hand, p75^+^ cells purified from adult human testes show the potential to differentiate into LCs in vivo when transplanted into the EDS-treated rat testes [69]. However, the expression level of p75 decreases in stages higher than 20-dpp in mouse testes [22,74]. Thus, p75 may not be appropriate as a candidate for a mouse SLC marker. As it is possible that stem cell markers are species-specific, more efforts are needed to identify markers specific for stem cells in mammalian testes.

## 5. Perspectives

As mentioned above, several reports have shown the presence of telocytes in adult testes in mammals, and dynamic behaviors of telocytes in vitro. However, several questions remain to be answered regarding telocytes in testes. Firstly, CD34 and PDGFRα (and also c-kit) have been used for markers of telocytes in testes, as well as in other various organs. However, the mesenchymal cells of testes in rodents and human have been shown to comprise some different populations, each with a distinct marker. Then, how about the relationship between the stem cell markers so far reported, and CD34 and/or PDGFRα? *Nes-GFP*^+^ cells derived from 7-dpp mouse testes show the expression of CD51, p75, and PDGFRα [66], and CD51^+^ cells express Nestin and PDGFRα in postnatal testes [67]. Combining these observations and ours, in that expressions of CD34, PDGFRα, and p75 highly overlap with each other in the postnatal testes [22], it may be reasonable to assume that a fairly high percentage of CD34^+^ mesenchymal cells express Nestin, CD51, and p75, though the percentage with such an overlap is unknown. Photographs showing the localization of CD90^+^ cells on the surface of freshly isolated seminiferous tubules indicate that the CD90^+^ cell population density in the interstitium is fairly high [68], such as that of CD34^+^ mesenchymal cells. It is, however, unknown how much of the CD90^+^ cell population overlaps with the CD34^+^ mesenchymal cell population, because the relationship between the population of mesenchymal CD34^+^PDGFRα^+^ cells and that of the other cell types has not been clearly determined [73]. Sc-RNAseq has shown that a cluster of the mesenchymal cell population expressing *Tcf21* isolated from adult mouse testes is heterogeneous, and that three of the five Tcf21^lin^ clusters express CD34, indicating that the CD34^+^ cell population is heterogeneous [72]. Thus, sc-RNAseq analysis of CD34^+^PDGFRα^+^ cells will be required to examine their heterogeneity, and if they are heterogeneous, it will further be required to find which population(s) is (are) telocytes. Secondly, morphological studies on telocytes have so far been performed mainly on adult testes [23,26,27,28], and there have been very few studies on telocytes in testes during development. Abe et al. [22] have shown that CD34 is expressed in CD31^−^ PDGFRα^+^ non-endothelial (mesenchymal) cells and CD31^+^ PDGFRα^−^ endothelial cells (the ratio is about 9:1) in mouse postnatal testes, while it is expressed only in CD31^+^PDGFRα^−^ endothelial cells in embryonic testes. On the other hand, Kuroda et al. [23] reported that a reticular network of CD34^+^ stromal cells is present between peritubular tissue and intertubular stroma in human fetuses. So, whether telocytes are present in embryonic testes in rodents and primates or not is an intriguing question, and if it is found that they are, this will raise further questions as to whether the telocytes are endothelial cells, mesenchymal cells, or other cell types, how they behave, and what their roles are in embryonic testes development. Thirdly, ALCs are reported to differentiate in postnatal testes from mesenchymal stem/progenitor cells and/or vascular pericytes, with various markers such as Nestin, CD90, CD51, p75, PDGFRα, and CD34, as mentioned above. When EDS is administered to adult rats/mice, ALCs regenerate after the deterioration of the resident ALCs. In both normal differentiation and regeneration, ALC differentiation starts with the extensive proliferation of mesenchymal stem/progenitor cells at a peritubular position. The progenitor cells and/or immature ALCs move toward the central interstitium, assemble to make clusters, and finally differentiate to mature ALCs, which are eventually enveloped by mesenchymal interstitial cells within their thin cytoplasmic extensions [4,5]. Hence, it may be interesting to observe morphological changes that may occur in telocytes during the processes of LC differentiation in normal testes, as well as of LC regeneration in EDS-treated rodent testes. Fourthly, studies on telocytes have been mainly concentrated on morphological aspects, and functional analyses are scarce. The morphology of telocytes may change dynamically through interactions with other surrounding cells and their milieu (hormones, growth factors, etc.) during development and in pathological disorders. To investigate the roles and functions of telocytes in spermatogenesis, an in vitro system would seem to be useful for analyzing the mechanisms underlying various phenomena. Our group has established an in vitro re-aggregate culture system in which seminiferous tubule-like structures and interstitial-like structures are reconstructed [37]. When dissociated testicular cells are deprived of interstitial cells and peritubular cells, and cultured, seminiferous tubule-like structures are not reconstructed, and instead, dysmorphic structures are formed [39]. However, when the fractionated interstitial cells and peritubular cells are added back to the dissociated cells deprived of the interstitial cells and peritubular cells, seminiferous tubule-like structures with interstitial-like structures are reconstructed, indicating that interstitial and peritubular cells are indispensable for the reconstruction of the seminiferous tubule-like structures from dissociated cells derived from neonatal mouse testes. Hence, this culture system represents one of the basic and invaluable systems for investigating the role of each cell type of interstitial and peritubular cells, such as CD34^+^PDGFRα^+^ mesenchymal cells (telocytes), LCs, PMCs, immune cells, and endothelial cells in the reconstruction of the seminiferous tubule-like structures. It may be interesting to eliminate a particular cell type; for example, CD34^+^PDGFRα^+^ telocytes, from the dissociated cells and perform re-aggregate culture to see what cell type(s) is (are) indispensable for the reconstruction. If an indispensable cell type is found, it will also be intriguing to modify some genes in that particular cell type, and to add these modified cells to the re-aggregate culture to clarify what genes play critical roles in the reconstruction. Abe et al. [41] have also shown the dynamic behaviors of CD34^+^ cells and ALCs in cell culture, revealing that CD34^+^ cells approach ALCs, extend telopodes, and plunge into the aggregate of ALCs (and CD34^+^ cells). They have also indicated that this phenomenon occurs via chemotaxis involving integrin α4β1–VCAM1 interaction. Analyses of interactions between telocytes in which some genes are knocked out, and other cell types, such as Sertoli cells, LCs, or PMCs in culture under various conditions, will reveal the regulatory mechanisms whereby telocytes play an important role in the formation and maintenance of elaborate testicular structures, and in regeneration after various pathological perturbations.

## Abbreviations

PMCs	peritubular myoid cells
ALCs	adult Leydig cells
FLCs	fetal Leydig cells
SLCs	stem cells of ALCs
HSD17β3	17β-hydroxysteroid dehydrogenase type 3
3β-HSD	3β-hydroxysteroid dehydrogenase
PDGFRα	platelet-derived growth factor receptor α
KSR	KnockOut Serum Replacement
EDS	ethane dimethane sulfonate

## References

[1] Kim Y., Capel B. (2006). Balancing the bipotential gonad between alternative organ fates: A new perspective on an old problem. Dev. Dyn..

[2] Svingen T., Koopman P. (2013). Building the mammalian testis: Origins, differentiation, and assembly of the component cell populations. Genes Dev..

[3] Ungewitter E.K., Yao H.H. (2013). How to make a gonad: Cellular mechanisms governing formation of the testes and ovaries. Sex. Dev..

[4] Habert R., Lejeune H., Saez J.M. (2001). Origin, differentiation and regulation of fetal and adult Leydig cells. Mol. Cell. Endocrinol..

[5] Mendis-Handagama S.M.L.C., Ariyaratne H.B.S. (2001). Differentiation of the adult Leydig cell population in the postnatal testis. Biol. Reprod..

[6] Shima Y., Matsuzaki S., Miyabayashi K., Otake H., Baba T., Kato S., Huhtaniemi I., Morohashi K.-I. (2015). Fetal Leydig cells persist as an androgen-independent subpopulation in the postnatal testis. Mol. Endocrinol..

[7] Haider S.G. (2004). Cell biology of Leydig cells in the testis. Int. Rev. Cytol..

[8] Teerds K.J., Huhtaniemi I.T. (2015). Morphological and functional maturation of Leydig cells: From rodent models to primates. Hum. Reprod. Update.

[9] Ye L., Li X., Li L., Chen H., Ge R.-S. (2017). Insights into the development of the adult Leydig cell lineage from stem Leydig cells. Front. Physiol..

[10] Shima Y. (2019). Development of fetal and adult Leydig cells. Reprod. Med. Biol..

[11] Shima Y., Miyabayashi K., Haraguchi S., Arakawa T., Otake H., Baba T., Matsuzaki S., Shishido Y., Akiyama H., Tachibana T. (2013). Contribution of Leydig and Sertoli cells to testosterone production in mouse fetal testes. Mol. Endocrinol..

[12] Cool J., DeFalco T., Capel B. (2012). Testis formation in the fetal mouse: Dynamic and complex de novo tubulogenesis. Wiley Interdiscip. Rev. Dev. Biol..

[13] Heinrich A., DeFalco T. (2020). Essential roles of interstitial cells in testicular development and function. Andrology.

[14] DeFalco T., Takahashi S., Capel B. (2011). Two distinct origins for Leydig cell progenitors in the fetal testis. Dev. Biol..

[15] Rotgers E., Jørgensen A., Yao H.H.-C. (2018). At the crossroads of fate-somatic cell lineage specification in the fetal gonad. Endocr. Rev..

[16] Chen H., Wang Y., Ge R.-S., Zirkin B.R. (2017). Leydig cell stem cells: Identification, proliferation and differentiation. Mol. Cell. Endocrinol..

[17] Shima Y., Miyabayashi K., Sato T., Suyama M., Ohkawa Y., Doi M., Okamura H., Suzuki K. (2018). Fetal Leydig cells dedifferentiate and serve as adult Leydig stem cells. Development.

[18] Gnessi L., Basciani S., Mariani S., Arizzi M., Spera G., Wang C., Bondjers C., Karlsson L., Betsholtz C. (2000). Leydig cell loss and spermatogenic arrest in platelet-derived growth factor (PDGF)-A–deficient mice. J. Cell Biol..

[19] Brennan J., Tilmann C., Capel B. (2003). *Pdgfr-*α mediates testis cord organization and fetal Leydig cell development in the XY gonad. Genes Dev..

[20] Gnessi L., Emidi A., Jannini E.A., Carosa E., Maroder M., Arizzi M., Ulisse S., Spera G. (1995). Testicular development involves the spatiotemporal control of PDGFs and PDGF receptors gene expression and action. J. Cell Biol..

[21] Basciani S., Mariani S., Spera G., Gnessi L. (2010). Role of platelet-derived growth factors in the testis. Endocr. Rev..

[22] Abe K., Kameyama H., Abe S.-I. (2022). CD34 is expressed in endothelial cells in embryonic testes and is additionally expressed in non-endothelial cells in postnatal mouse testes. Zool. Sci..

[23] Kuroda N., Nakayama H., Miyazaki E., Hayashi Y., Toi M., Hiroi M., Enzan H. (2004). Distribution and role of CD34-positive stromal cells and myofbroblasts in human normal testicular stroma. Histol. Histopathol..

[24] Popescu L.M., Faussone-Pellegrini M.-S. (2010). TELOCYTES—A case of serendipity: The winding way from Interstitial Cells of Cajal (ICC), via Interstitial Cajal-like Cells (ICLC) to TELOCYTES. J. Cell. Mol. Med..

[25] Yang P., Ahmad N., Hunag Y., Ullah S., Zhang Q., Waqas Y., Liu Y., Li Q., Hu L., Chen Q. (2015). Telocytes: Novel interstitial cells present in the testis parenchyma of the Chinese soft-shelled turtle *Pelodiscus sinensis*. J. Cell. Mol. Med..

[26] Marini M., Rosa I., Guasti D., Gacci M., Sgambati E., Ibba-Manneschi L., Manetti M. (2018). Reappraising the microscopic anatomy of human testis: Identification of telocyte networks in the peritubular and intertubular stromal space. Sci. Rep..

[27] Liu Y., Liang Y., Wang S., Tarique I., Vistro W.A., Zhang H., Haseeb A., Gandahi N.S., Iqbal A., An T. (2019). Identification and characterization of telocytes in rat testis. Aging.

[28] Pawlicki P., Hejmej A., Milon A., Lustofin K., Płachno B.J., Tworzydlo W., Gorowska-Wojtowicz E., Pawlicka B., Kotula-Balak M., Bilinska B. (2019). Telocytes in the mouse testicular interstitium: Implications of G-protein-coupled estrogen receptor (GPER) and estrogen-related receptor (ERR) in the regulation of mouse testicular interstitial cells. Protoplasma.

[29] Bei Y., Wang F., Yang C., Xiao J. (2015). Telocytes in regenerative medicine. J. Cell. Mol. Med..

[30] Manova K., Nocka K., Besmer P., Bachvarova R.F. (1990). Gonadal expression of *c-kit* encoded at the *W* locus of the mouse. Development.

[31] Yoshinaga K., Nishikawa S., Ogawa M., Hayashi S., Kunisada T., Fujimoto T., Nishikawa S. (1991). Role of c-kit in mouse spermatogenesis: Identification of spermatogonia as a specific site of c-kit expression and function. Development.

[32] Aumuller G., Schulze C., Viebahn C. (1992). Intermediate filaments in Sertoli cells. Microsc. Res. Tech..

[33] Alves-Lopes J.P., Stukenborg J.-B. (2018). Testicular organoids: A new model to study the testicular microenvironment in vitro?. Hum. Reprod. Update.

[34] Richer G., Baert Y., Goossens E. (2020). In-vitro spermatogenesis through testis modelling: Towards the generation of testicular organoids. Andrology.

[35] Sakib S., Goldsmith T., Voigt A., Dobrinski I. (2020). Testicular organoids to study cell–cell interactions in the mammalian testis. Andrology.

[36] Cham T.-C., Chen X., Honaramooz A. (2021). Current progress, challenges, and future prospects of testis organoids. Biol. Reprod..

[37] Zhang J., Hatakeyama J., Eto K., Abe S.-I. (2014). Reconstruction of a seminiferous tubule-like structure in a 3 dimensional culture system of re-aggregated mouse neonatal testicular cells within a collagen matrix. Gen. Comp. Endocrinol..

[38] Zhang X., Wang L., Zhang X., Ren L., Shi W., Tian Y., Zhu J., Zhang T. (2017). The use of KnockOut serum replacement (KSR) in three dimensional rat testicular cells co-culture model: An improved male reproductive toxicity testing system. Food Chem. Toxicol..

[39] Abe S.-I., Abe K., Zhang J., Harada T., Mizumoto G., Oshikawa H., Akiyama H., Shimamura K. (2017). Roles of CD34^+^ cells and ALK5 signaling in the reconstruction of seminiferous tubule-like structures in 3-D re- aggregate culture of dissociated cells from neonatal mouse testes. PLoS ONE.

[40] Miles D.C., Wakeling S.I., Stringer J.M., van den Bergen J.A., Wilhelm D., Sinclair A.H., Western P.S. (2013). Signaling through the TGF beta-activin receptors ALK4/5/7 regulates testis formation and male germ cell development. PLoS ONE.

[41] Abe K., Kon S., Kameyama H., Zhang J., Morohashi K.-i., Shimamura K., Abe S.-I. (2021). VCAM1-α4β1 integrin interaction mediates interstitial tissue reconstruction in 3-D re-aggregate culture of dissociated prepubertal mouse testicular cells. Sci. Rep..

[42] Cook-Mills J.M., Marchese M.E., Abdala-Valencia H. (2011). Vascular cell adhesion molecule-1 expression and signaling during disease: Regulation by reactive oxygen species and antioxidants. Antioxid. Redox Signal..

[43] Kong D.-H., Kim Y.K., Kim M.R., Jang J.H., Lee S. (2018). Emerging roles of vascular cell adhesion molecule-1 (VCAM-1) in immunological disorders and cancer. Int. J. Mol. Sci..

[44] Kitani A., Nakashima N., Izumihara T., Inagaki M., Baoui X., Yu S., Matsuda T., Matsuyama T. (1998). Soluble VCAM-1 induces chemotaxis of Jurkat and synovial fluid T cells bearing high affinity very late antigen-4. J. Immunol..

[45] Sidney L.E., Branch M.J., Dunphy S.E., Dua H.S., Hopkinson A. (2014). Concise Review: Evidence for CD34 as a common marker for diverse progenitors. Stem Cells.

[46] Cretoiu S.M., Popescu L.M. (2014). Telocytes revisited. Biomol. Concepts.

[47] Cretoiu D., Radu B.M., Banciu A., Banciu D.D., Cretoiu S.M. (2017). Telocytes heterogeneity: From cellular morphology to functional evidence. Semin. Cell Dev. Biol..

[48] Díaz-Flores L., Gutiérrez R., García M.P., Gayoso S., Gutiérrez E., Díaz-Flores L., Carrasco J.L. (2020). Telocytes in the normal and pathological peripheral nervous system. Int. J. Mol. Sci..

[49] Díaz-Flores L., Gutiérrez R., García M.P., Sáez F.J., Díaz- Flores L., Valladares F., Madrid J.F. (2014). CD34^+^ stromal cells/fibroblasts/fibrocytes/telocytes as a tissue reserve and a principal source of mesenchymal cells. Location, morphology, function and role in pathology. Histol. Histopathol..

[50] Díaz-Flores L., Gutiérrez R., Díaz-Flores L., Goméz M.G., Sáez F.J., Madrid J.F. (2016). Behaviour of telocytes during physiopathological activation. Semin. Cell Dev. Biol..

[51] Rosa I., Marini M., Manetti M. (2021). Telocytes: An emerging component of stem cell niche microenvironment. J. Histochem. Cytochem..

[52] Díaz-Flores L., Gutiérrez R., García M.P., González M., Sáez F.J., Aparicio F., Díaz-Flores L., Madrid J.F. (2015). Human resident CD34^+^ stromal cells/telocytes have progenitor capacity and are a source of αSMA^+^ cells during repair. Histol. Histopathol..

[53] Kumar D.L., DeFalco T. (2018). A perivascular niche for multipotent progenitors in the fetal testis. Nat. Commun..

[54] Sawamoto K., Nakao N., Kakishita K., Ogawa Y., Toyama Y., Yamamoto A., Yamaguchi M., Mori K., Goldman S.A., Itakura T. (2001). Generation of dopaminergic neurons in the adult brain from mesencephalic precursor cells labeled with a *nestin-GFP* transgene. J. Neurosci..

[55] Mendez-Ferrer S., Michurina T.V., Ferraro F., Mazloom A.R., MacArthur B.D., Lira S.A., Scadden D.T., Ma’ayan A., Enikolopov G.N., Frenette P.S. (2010). Mesenchymal and haematopoietic stem cells form a unique bone marrow niche. Nature.

[56] Bernal A., Arranz L. (2018). Nestin-expressing progenitor cells: Function, identity and therapeutic implications. Cell. Mol. Life Sci..

[57] Kitamura K., Yanazawa M., Sugiyama N., Miura H., Iizuka-Kogo A., Kusaka M., Omichi K., Suzuki R., Kato-Fukui Y., Kamiirisa K. (2002). Mutation of *ARX* causes abnormal development of forebrain and testes in mice and X-linked lissencephaly with abnormal genitalia in humans. Nat. Genet..

[58] Miyabayashi K., Katoh-Fukui Y., Ogawa H., Baba T., Shima Y., Sugiyama N., Kitamura K., Morohashi K.-I. (2013). Aristaless related homeobox gene, *Arx*, is implicated in mouse fetal Leydig cell differentiation possibly through expressing in the progenitor cells. PLoS ONE.

[59] Tsai S.Y., Tsai M.J. (1997). Chick ovalbumin upstream promoter-transcription factors (COUP-TFs): Coming of age. Endocr. Rev..

[60] Ariyaratne S., Kim I., Mills N., Mason I., Mendis-Handagama C. (2003). Effects of ethane dimethane sulfonate on the functional structure of the adult rat testis. Arch. Androl..

[61] Davidoff M.S., Middendorff R., Enikolopov G., Riethmacher D., Holstein A.F., Muller D. (2004). Progenitor cells of the testosterone-producing Leydig cells revealed. J. Cell Biol..

[62] Ge R.-S., Dong Q., Sottas C.M., Papadopoulos V., Zirkin B.R., Hardy M.P. (2006). In search of rat stem Leydig cells: Identification, isolation, and lineage-specific development. Proc. Natl. Acad. Sci. USA.

[63] Stanley E., Lin C.-Y., Jin S., Liu J., Sottas C.M., Ge R.-S., Zirkin B.R., Chen H. (2012). Identification, proliferation, and differentiation of adult Leydig stem cells. Endocrinology.

[64] Qin J., Tsai M.-J., Tsai S.Y. (2008). Essential roles of COUP-TFII in Leydig cell differentiation and male fertility. PLoS ONE.

[65] Kilcoyne K.R., Smith L.B., Atanassova N., Macpherson S., McKinnell C., van den Driesche S., Jobling M.S., Chambers T.J.G., De Gendt K., Verhoeven G. (2014). Fetal programming of adult Leydig cell function by androgenic effects on stem/progenitor cells. Proc. Natl. Acad. Sci. USA.

[66] Jiang M.H., Cai B., Tuo Y., Wang J., Zang Z.J., Tu X., Gao Y., Su Z., Li W., Li G. (2014). Characterization of Nestin-positive stem Leydig cells as a potential source for the treatment of testicular Leydig cell dysfunction. Cell Res..

[67] Zang Z.J., Wang J., Chen Z., Zhang Y., Gao Y., Su Z., Tuo Y., Liao Y., Zhang M., Yuan Q. (2017). Transplantation of CD51^+^ stem Leydig cells: A new strategy for the treatment of testosterone deficiency. Stem Cells.

[68] Li X., Wang Z., Jiang Z., Guo J., Zhang Y., Li C., Chung J., Folmer J., Liu J., Lian Q. (2016). Regulation of seminiferous tubule-associated stem Leydig cells in adult rat testes. Proc. Natl. Acad. Sci. USA.

[69] Zhang M., Wang J., Deng C., Jiang M.H., Feng X., Xia K., Li W., Lai X., Xiao H., Ge R.-S. (2017). Transplanted human p75-positive stem Leydig cells replace disrupted Leydig cells for testosterone production. Cell Death Dis..

[70] Stévant I., Neirijnck Y., Borel C., Escoffier J., Smith L.B., Antonarakis S.E., Dermitzakis E.T., Nef S. (2018). Deciphering cell lineage specification during male sex determination with single-cell RNA sequencing. Cell Rep..

[71] Green C.D., Ma Q., Manske G.L., Shami A.N., Zheng X., Marini S., Moritz L., Sultan C., Gurczynski S.J., Moore B.B. (2018). A comprehensive roadmap of murine spermatogenesis defined by single-cell RNA-Seq. Dev. Cell.

[72] Shen Y., Shami A.N., Moritz L., Larose H., Manske G.L., Ma Q., Zheng X., Sukhwani M., Czerwinski M., Sultan C. (2021). TCF21^+^ mesenchymal cells contribute to testis somatic cell development, homeostasis, and regeneration in mice. Nat. Commun..

[73] Guan X., Chen P., Ji M., Wen X., Chen D., Zhao X., Huang F., Wang J., Shao J., Xie J. (2022). Identification of rat testicular Leydig precursor cells by single-cell-RNA-sequence analysis. Front. Cell Dev. Biol..

[74] Russo M.A., Giustizieri M.L., Favale A., Fantini M.C., Campagnolo L., Konda D., Germano F., Farini D., Manna C., Siracusa G. (1999). Spatiotemporal patterns of expression of neurotrophins and neurotrophin receptors in mice suggest functional roles in testicular and epididymal morphogenesis. Biol. Reprod..

